# Way More than the Sum of Their Parts: From Statistical to Structural Mixtures

**DOI:** 10.3390/e28010111

**Published:** 2026-01-16

**Authors:** James P. Crutchfield

**Affiliations:** Complexity Sciences Center and Physics and Astronomy Department, University of California at Davis, One Shields Avenue, Davis, CA 95616, USA; chaos@ucdavis.edu

**Keywords:** epsilon-machine, nonergodicity, causal state, multistationary, excess entropy, statistical complexity, hierarchy, transients

## Abstract

We show that mixtures comprising multicomponent systems typically are much more structurally complex than the sum of their parts; sometimes, infinitely more complex. We contrast this with the more familiar notion of statistical mixtures, demonstrating how statistical mixtures miss key aspects of emergent hierarchical organization. This leads us to identify a new kind of structural complexity inherent in multicomponent systems and to draw out broad consequences for system ergodicity.

## 1. Introduction

Multicomponent systems typically are much more structurally complex than the collection of their parts, even infinitely more so. This should be contrasted with statistical mixtures—such as arise in the Gibbs Paradox of thermodynamics (Sections 2–3 in [1]), where gases of distinct molecular species exhibit only a modest entropy increase upon formation due to the uncertainty in which species one has in hand. This contrast demonstrates how the ansatz of statistical mixtures misses key aspects of hierarchical organization. The result, as we show, is an awareness of a new kind of structural complexity of composite systems.

The development here focuses on the theoretical core of this basic phenomenon, arguing that it is, in fact, quite commonplace. To appreciate this, it will be helpful to address the motivating issues upfront.

The multicomponent systems of interest are found in several different domains, including the entropy of mixing in thermodynamics [2,3], the change point problem in statistics [4], the attractor-basin portrait of a dynamical system [5], Smale’s basic sets [6,7], spatially extended systems with multiple local attractors [8], chaotic crystallography [9,10], evolutionary dynamics [11], and adaptive and learning systems with memory. More recently, nonergodicity has been broadly implicated in, for example, computation theory [12], learning theory [13], and the complex behaviors of social and economic systems [14,15].

We introduce the concept of hidden multistationary processes to capture what is common across these domains—a system comprising multiple locally competing behaviors and structures. The basic idea can be appreciated within an experimental paradigm: multistationarity models repeated experimental trials in which different initial conditions lead to statistically distinct behaviors. When we wish to emphasize their structure, we refer to a *multicomponent process*; when emphasizing the statistical consequences, we refer to a *multistationary process*.

In short, one goal is to provide a tractable model that quantitatively captures what is common among these domains while providing an architectural, high-level view of the state-space organization of behaviors. In particular, we would like to analyze how unpredictable and how structurally complex hidden multistationary processes are when given their components, whose unpredictability and complexity we know. Another goal is that the approach be constructive, allowing one to quantitatively determine essential properties and to determine precisely what gives rise to the emergent global complexity.

The development proceeds as follows: It first reviews statistical mixtures, briefly recalling stochastic processes, information theory, structural complexity, and mixed-state processes. It then introduces the theory and construction of hidden multistationary processes. This includes a canonical minimal representation of hidden multistationary processes and a method to analyze their ergodic decompositions that determines how the latter affect information measures.

The sections following this explore a number of examples, going from the simplest cases and familiar structured stationary component processes to the *Mother of All Processes* that subsumes them all. Taken altogether, these illustrate a new kind of structural hierarchy and make plain how infinite complexity naturally emerges. The development concludes by drawing out parallels with related results and consequences in nonequilibrium thermodynamics and machine learning.

## 2. Background

To get started, we give a minimal summary of the required background—a summary that assumes familiarity with computational mechanics [16,17] and with information theory for complex systems [18,19].

### 2.1. Processes

A process, denoted P, is specified by the joint distribution $P({\overset{\leftarrow }{X}}_{t},{\vec{X}}_{t})$ over its chain of random variables $\ldots X_{-1}X_{0}X_{1}\ldots$. We view P as a *communication channel* with a fixed input distribution $P({\overset{\leftarrow }{X}}_{t})$: It transmits information from the *past*${\overset{\leftarrow }{X}}_{t}=\ldots X_{t-3}X_{t-2}X_{t-1}$ to the *future* ${\vec{X}}_{t}=X_{t}X_{t+1}X_{t+2}\ldots$ by storing it in the present. $X_{t}$ denotes the discrete random variable at time *t* taking on values *x* from a discrete alphabet A. And $X_{t}^{\ell }=X_{t}X_{t+1}\ldots X_{t+\ell -1}$ is the block of *ℓ* random variables starting at time *t*. A particular realization is denoted using lowercase: $X_{t}^{\ell }=x_{t}^{\ell }\in A^{\ell }$. Often, we simply refer to a particular sequence $w=x_{0}x_{1}\ldots x_{\ell -1}$, $x_{i}\in A$, as a *word*. If we have a symbol *x* and a word *w*, we form a new word by concatenation, e.g., wx or xw.

### 2.2. Information

Given a process, we form the block distributions $\left\{P\left(X_{t}^{\ell }\right):\mathrm{for}\;\mathrm{all}\;t\;\mathrm{and}\;\ell \right\}$ by marginalizing the given joint distribution

(1) $$\begin{array}{c} P\left(X_{t}^{\ell }\right)=\sum_{\left\{{\overset{\leftarrow }{x}}_{t},{\vec{x}}_{t+\ell }\right\}}P\left({\overset{\leftarrow }{x}}_{t},{\vec{x}}_{t+\ell }\right). \end{array}$$

(We ignore here the measure-theoretic construction of cylinder sets and their measures; for background, see Ref. [20] and references therein.) A stationary process is one for which $P\left(X_{t}^{\ell }\right)=P\left(X_{0}^{\ell }\right)$ for all *t* and *ℓ*. For a stationary process, we drop the time index and thereby have the family of *word distributions* $P(X^{\ell })$ that completely characterizes the process.

The amount of Shannon information in words is measured by the *block entropy*,

(2) $$\begin{array}{c} H\left(\ell \right)=H[P\left(X^{\ell }\right)], \end{array}$$

where $H\left[P\left(Y\right)\right]=-\sum_{\left\{y\right\}}P\left(y\right)\log_{2}P\left(y\right)$ is the Shannon entropy of the random variable *Y*. A process’ information production is given by its *entropy rate*,

(3) $$\begin{array}{c} h_{\mu }=\lim_{\ell \to \infty }\frac{H(\ell )}{\ell }, \end{array}$$

where μ refers to the measure over infinite sequences and so, too, to the word probabilities P(w). It is often used to measure a process’ degree of unpredictability.

At a minimum, a good predictor—denote this model’s state random variables $\hat{R}$—must capture *all* of a process’ *excess entropy* E [19]—all of the information shared between past and future: $E=I[\overset{\leftarrow }{X};\vec{X}]$. Here, I[Y;Z] is the mutual information between variables *Y* and *Z*. That is, for a good predictor $\hat{R}$: $E=I[\hat{R};\vec{X}]$.

These quantities are closely related. In particular, for finitary processes, those with E<∞, the block entropy has the linear asymptotic behavior,

$$\begin{array}{c} H\left(\ell \right)\propto_{\ell \to \infty }E+h_{\mu }\ell . \end{array}$$

More precisely,

(4) $$\begin{array}{c} E=\lim_{\ell \to \infty }\left[H\left(\ell \right)-h_{\mu }\ell \right]. \end{array}$$

This shows that E controls the convergence of entropy rate estimates $h_{\mu }\left(\ell \right)=H\left(\ell \right)-H\left(\ell -1\right)$. In fact, for time-series processes, E can also be defined in terms of entropy convergence,

(5) $$\begin{array}{c} E=\sum_{\ell =1}^{\infty }\left[h_{\mu }\left(\ell \right)-h_{\mu }\right]. \end{array}$$

An analogous quantity that controls the block entropy convergence to the linear asymptote is the *transient information*,

(6) $$\begin{array}{c} T=\sum_{\ell =0}^{\infty }\left[E+h_{\mu }\ell -H\left(\ell \right)\right]. \end{array}$$

T measures the average amount of information an observer must extract in order to know a process’ internal state (for a review of these and related informations, see Ref. [19]).

### 2.3. Structure

We refer to a model of a process—a particular choice of $\hat{R}$—as a *presentation*. Note that building a model of a process is more demanding than developing a prediction scheme, since one wishes to go beyond sequence statistics to express a process’ mechanisms and internal organization.

To do this, we first recall that a process’ communication channel is determined by the conditional distributions $P({\vec{X}}_{t}|{\overset{\leftarrow }{X}}_{t})$. Based on this, computational mechanics introduced an equivalence relation $\overset{\leftarrow }{x}{∼}_{\epsilon }{\overset{\leftarrow }{x}}^{\prime }$ that groups all of a process’ histories that give rise to the same prediction. This results in constructing a map $\epsilon :\overset{\leftarrow }{X}\to S$ from all pasts (finite and infinite) to *causal states* defined by

(7) $$\begin{array}{c} \epsilon \left({\overset{\leftarrow }{x}}^{\ell }\right)=\left\{{\overset{\leftarrow }{x}}^{\prime \ell }:P\left(\vec{X}|{\overset{\leftarrow }{x}}^{\ell }\right)=P\left(\vec{X}|{\overset{\leftarrow }{x}}^{\prime \ell }\right)\right\}, \end{array}$$

where ${\overset{\leftarrow }{x}}^{\ell },{\overset{\leftarrow }{x}}^{\prime \ell }\in A^{\ell }$. In other words, a process’ *finite-history* causal states are equivalence classes—$S^{\ell }=P\left({\overset{\leftarrow }{X}}^{\ell },\vec{X}\right)/\;\;{∼}_{\epsilon }$—that partition the space ${\overset{\leftarrow }{X}}^{\ell }$ of pasts into sets which are predictively equivalent. The causal states, then, are the collection across all past lengths: $S=\cup_{\ell =0}^{\infty }S^{\ell }$. These consist of *recurrent* and *transient* causal states that are visited with positive or vanishing probability, respectively.

With the causal states in hand, one determines the causal-state to causal-state transitions,

$$\begin{array}{c} \{T_{\sigma ,\sigma^{\prime }}^{\left(x\right)}:x\in A,\sigma ,\sigma^{\prime }\in S\}. \end{array}$$

The resulting model *M*, consisting of the causal states and transitions, is called the process’ *ϵ-machine* [21],

(8) $$\begin{array}{c} M\left(P\right)\equiv \left\{S,T^{\left(x\right)}:x\in A\right\}. \end{array}$$

Informally, a process is *ergodic* if its statistics can be estimated from a single realization that is sufficiently long. If P is ergodic, then M(P)’s recurrent causal states are strongly connected and their asymptotic invariant distribution π=P(S) is unique and given by π=πT, where $T=\sum_{x\in A}T^{\left(x\right)}$.

As described, an ϵ-machine is obtained from a process, but one can also simply define an ϵ-machine and consider its generated process. We will use both notions in the following, as they are equivalent [22]. But why should one use the ϵ-machine presentation of a process in the first place?

The main takeaway from computational mechanics is that out of all optimally predictive models $\hat{R}$ resulting from a partition of the past—those such that $E=I[\hat{R};\vec{X}]$—the ϵ-machine captures the amount of information that a process stores—the *statistical complexity* $C_{\mu }\equiv H\left[P\left(S\right)\right]$. The excess entropy E—the information explicitly observed in sequences—is only a lower bound on the information $C_{\mu }$ that a process stores [21]: $E\leq C_{\mu }$. The difference $\chi =C_{\mu }-E$, called the *crypticity*, measures how the process hides its internal state information from an observer [23].

A process’ ϵ-machine is its minimal unifilar presentation. It is unique for the process. Moreover, it allows a number of the process’ complexity measures to be directly and efficiently calculated [24]. The latter include the process’ entropy rate, excess entropy, statistical complexity, and crypticity. In short, a process’ ϵ-machine captures all of its informational and structural properties.

## 3. Mixed State Operator

Given an ϵ-machine *M*, its recurrent causal states can be treated as a standard basis $\left\{e_{j}\right\}$ in a vector space. Then, any distribution μ=P(S) over the states is a linear combination: $\mu =\sum_{j}c_{j}e_{j}$. Following Ref. [17], these distributions are called *mixed states*. For an *k*-state ϵ-machine, the mixed-state space is a k−1-dimensional simplex $\Delta^{k-1}$, as the distributions $\mu \in \Delta^{k-1}$ are normalized.

Consider a special subset of mixed states. Define μ(w) as the distribution over *M*’s states induced after observing sequence $w=x_{0}\ldots x_{\ell -1}$, *M* having started with state distribution π,

(9) $$\begin{array}{cc} \mu_{\pi }\left(w\right) & \equiv P(S_{\ell }|X_{0}^{\ell }=w,S_{0}∼\pi )\\ \; & =\frac{P(X_{0}^{\ell }=w,S_{\ell },S_{0}∼\pi )}{P(X_{0}^{\ell }=w,S_{0}∼\pi )}\\ \; & =\frac{\pi T^{\left(w\right)}}{\pi T^{\left(w\right)}1}, \end{array}$$

where **1** is a column vector of 1s and $T^{\left(w\right)}=T^{\left(x_{\ell -1}\right)}\ldots T^{\left(x_{0}\right)}$. Here, the notation X∼P serves to indicate that random variable *X* is governed by distribution *P*.

The last line gives the mixed-state $\mu_{\pi }\left(w\right)$ directly in terms of the initial state distribution π and *M*’s transition matrices. One interpretation is that $\mu_{\pi }\left(w\right)$ represents an observer’s best guess as to the process’ causal-state distribution given that it saw word *w* and knows both the process’ ϵ-machine and the initial distribution π. Occasionally in the following, it will be noted that π refers not to the initial distribution but to another.

To determine the set of mixed states allowed by a process, we simply calculate the set $\left\{\mu_{\pi }\left(w\right)\right\}$ of distinct $\mu_{\pi }\left(w\right)$ for all words $w\in A^{*}$. This is most directly achieved by enumerating *w* in lexicographic order, e.g., for a binary alphabet, successively choosing w∈{λ,0,1,00,01,10,11,…}. Here, λ is the null word. As we will see, the mixed-state set can be finite or infinite.

If we consider the entire set of mixed states, then we construct a presentation of the process by specifying the transition matrices $\left\{T^{x}:\mu_{\pi }\left(w\right)\to \mu_{\pi }\left(wx\right)\right\}$,

(10) $$\begin{array}{cc} P(x,\mu_{\pi }\left(wx\right)|\mu_{\pi }\left(w\right)) & \equiv \frac{P(wx|S_{0}∼\pi )}{P(w|S_{0}∼\pi )}\\ \; & =\mu_{\pi }\left(w\right)T^{\left(x\right)}1\;\;1. \end{array}$$

Note that many words can induce the same mixed state.

It is useful to define a corresponding operator U that acts on a machine *M*, returning its *mixed-state presentation*$U_{\pi }\left(M\right)=\left\{\left\{T^{x}\right\},\left\{\mu_{\pi }\left(w\right)\right\}\right\}$ under initial distribution π. The examples to follow shortly illustrate how mixed states and $U_{\pi }\left(M\right)$ are calculated.

## 4. Constructing Hidden Multistationary Processes

Recall that a hidden multistationary nonergodic process is one that evolves, across successive realizations, to statistically distinct long-term behaviors. We now introduce our model of this by giving a construction procedure. This, in effect, defines what we mean by multistationary. We then develop several basic properties and analyze in detail a series of example constructions to illustrate them and their ergodic decompositions.

The main tool used to construct a hidden multistationary process is the mixed-state operator $U_{\pi }$. We show that this results in a canonical presentation of a given set of stationary components. This is the multistationary process’ ϵ-machine.

**Definition** **1.** *A* hidden multistationary process *(HMSP) is defined by the presentation determined via the following procedure:*

1. *Identify an indexed family of distinct component stationary ergodic processes ${\left\{P^{i}\right\}}_{i\in I}$, where I is a finite or countable index set. Each is specified by its ϵ-machine presentation $M^{i}=M\left(P^{i}\right)$. The ϵ-machines consist only of their recurrent states $S^{i}$ that, due to ergodicity, form a single, strongly connected set.*
2. *Specify the component* mixture distribution *π—the probability with which each will be visited (sampled),*
  (11) $$\begin{array}{c} \pi^{i}=P\left(M^{i}\right). \end{array}$$
3. *Finally, calculate the mixed-state presentation of the multistationary process,*
  (12) $$\begin{array}{c} M=U_{\pi }\left(\underset{i\in I}{⨆}M^{i}\right), \end{array}$$
  *where we take the nonoverlapping set of the measure semi-groups [25] specified by the component ϵ-machines. In this way, M’s states and transitions are determined from the component ϵ-machines and the mixture distribution π.*

The HMSP *M*, the result of the construction, determines the transient portion of a nonergodic ϵ-machine. *M*’s recurrent components are essentially the same as those ($M^{i}$’s) of the original component stationary processes $P^{i}$. That is to say, what is new in *M* is the set of transient causal states.

Note that this construction is a stochastic analog of building recognizers for multiregular formal languages [26].

## 5. The Multistationary ϵ-Machine

With the background and definitions set, we are ready to explore the properties of multistationary nonergodic processes. We first establish the structural properties of their ϵ-machine presentations and then their informational properties via ergodic decompositions of various complexity measures.

Each component $M^{i}=\left\{S^{i},T_{i}^{\left(x\right)},x\in A^{i}\right\}$, considered as generating its own process $P^{i}$, has a stationary distribution $p^{i}$ over its states,

$$\begin{array}{c} p_{j}^{i}=P\left(S_{j}\right),\;S_{j}\in S^{i}. \end{array}$$

We will also write this as a vector over the multistationary process’ recurrent states, when we have a finite number of components,

$$\begin{array}{c} \pi =\left[\pi^{1}\left(p_{1}^{1}\ldots p_{j_{1}}^{1}\right)\ldots \pi^{k}\left(p_{1}^{k}\ldots p_{j_{k}}^{k}\right)\right], \end{array}$$

where k=|I| and $j_{i}=\left|S^{i}\right|$. The stationary state distribution $\pi_{ij}$ for the multistationary process generated by *M* is, then

(13) $$\begin{array}{c} \pi_{ij}=\pi^{i}\cdot p_{j}^{i}. \end{array}$$

Consider the following properties of a multistationary process as just defined. The proofs of these results closely follow those of Theorem 1, Lemma 7, and Theorem 2 of Section 4 in Ref. [27]. One simply groups together the states and transitions of the nonergodic mixture component machines and applies Ref. [27]’s methods to these new (larger) sets of the composite machine. Reference [27]’s proof steps, then apply directly to obtain the results claimed. Indeed, many of the results for ϵ-machines in Ref. [27] carry over to the ϵ-machines for multistationary processes.

To help motivate the construction and rationale of multistationary ϵ-machines, the following describes their properties with short outlines of the arguments. The claims themselves, though, are stated here only as conjectures, leaving to a sequel the formal development and proofs. With this noted, for each of the example cases in Section 7 below, the properties can be verified by hand.

**Lemma** **1** (Stationarity).
*The state distribution $\pi_{ij}$ is stationary.*

**Proof.**  This follows from realizing that the recurrent portion of *M*’s transition matrix is block diagonal. That is, asymptotically the components are independent, and, by assumption, the component distributions are invariant. □

**Hypothesis** **1** (Unifilarity).
*The hidden multistationary process machine M is unifilar.*

**Remark** **1.** *This would follow by adapting Lemma 5 (ϵ-Machines Are Deterministic) of Ref. [27] to the composite machine’s mixed-state presentation.*

**Hypothesis** **2** (Minimality).
*The hidden multistationary process machine M is minimal.*

**Remark** **2.** *This would follow by adapting Theorem 2 (Causal States Are Minimal) of Ref. [27] to the composite machine’s mixed-state presentation. Recall that the latter is determined from each component’s ϵ-machine, which is minimal.*

**Hypothesis** **3** (Uniqueness).
*The hidden multistationary process machine M is unique.*

**Remark** **3.** *This would follow by adapting Theorem 3 (Causal States Are Unique) of Ref. [27] to the composite machine’s mixed-state presentation.*

As noted, the relevant definitions and proofs of these closely follow those given for ϵ-machines generally, as in Ref. [27], and will be the subject of a sequel.

**Hypothesis** **4.** *The mixed state operator applied to a mixture of (finite, ergodic) ϵ-machines produces an ϵ-machine. That is, the ϵ-machine for the hidden multistationary process generated by*

(14) $$\begin{array}{c} M=U\left(\underset{i\in I}{⨆}M^{i}\right) \end{array}$$

*is an ϵ-machine.*

**Remark** **4.** *This would follow from the preceding claims.*

*Remark*: Constructing HMSPs in this way, one could start with other classes of presentation for the ergodic component processes, such as nonunifilar presentations—i.e., generic HMMs. However, the resulting *M* need not be an ϵ-machine. And, as a consequence, one could not directly calculate from such an *M* the various complexity measures nor, lacking minimality, draw structural conclusions about its architecture. This is one reason why we choose to specify the component processes using ϵ-machine presentations. Limiting the current construction to ergodic components specified by finite-state ϵ-machine presentations serves to simplify the discussion and highlight our main results.

However, lifting these various restrictions or generalizing the previous properties to address them would be a fruitful effort, giving a much broader characterization of the complexity of multistationary processes.

So, from here on out we assume the ergodic components are ϵ-machines and ask what properties hold for the multistationary processes so constructed. We build processes consisting of either a finite number or countably infinite number of components.

## 6. Ergodic Decompositions

Since we are given the component processes $\left\{P^{i},i\in I\right\}$, what can we say about the resulting multistationary process generated by *M*? A first step develops various kinds of ergodic decomposition that attempt to predict *M*’s properties in terms of its ergodic components’ properties. The basic question has a very long history in ergodic and information theories. The reader is referred to the review given in Ref. [28]. Our approach here is, on the one hand, to briefly give a flavor of several ergodic decompositions and, on the other, to compensate for that lack of rigor, by analyzing in detail a number of concrete examples.

The word distribution $P(X^{\ell })$ for $M=U\left({⊔}_{i\in I}M^{i}\right)$ is given by

(15) $$\begin{array}{c} P\left(X^{\ell }\right)=\sum_{i\in I}\pi^{i}P\left(X^{\ell }|M^{i}\right). \end{array}$$

That is, for word *w*,

(16) $$\begin{array}{c} P\left(w\right)=\sum_{i\in I}\pi^{i}P_{i}\left(w\right), \end{array}$$

where $P_{i}\left(w\right)$ denotes the probability that $P^{i}$ generates *w*.

Quantitatively, the HMSP’s block entropy is upper bounded by the component block entropies,

(17) $$\begin{array}{cc} H(\ell ) & =H\left[P(X^{\ell })\right]\\ \; & =H\left[\sum_{i\in I}\pi^{i}P\left(X^{\ell }|M^{i}\right)\right]\;\\ \; & \leq \sum_{i\in I}\pi^{i}H\left[P(X^{\ell }|M^{i})\right]\\ \; & =\sum_{i\in I}\pi^{i}H^{i}\left(\ell \right), \end{array}$$

where the second-to-last step employs Jensen’s inequality [18] and $H^{i}\left(\ell \right)$ is component $P^{i}$’s block entropy.

A more insightful upper bound, though, is developed by first imagining that the sequences generated by the ergodic components do not overlap—for example, the $P^{i}$s have disjoint alphabets $A^{i}$. Then we define an indicator function *f* of the process and an associated random variable θ: $\theta =f(X^{\ell })=i$, if $X^{\ell }\in A_{i}^{\ell }$. We have

$$\begin{array}{cc} H\left[X^{\ell }\right]\; & =H\left[X^{\ell },f\left(X^{\ell }\right)\right]\\ \; & =H\left[\theta \right]+H\left[X^{\ell }|\theta \right]\\ \; & =H\left[\theta \right]+\sum_{i\in I}P\left(\theta =i\right)H\left[X^{\ell }|\theta =i\right]\\ \; & =H\left[\pi \right]+\sum_{i\in I}\pi^{i}H\left[X^{\ell }|M^{i}\right]\\ \; & =H\left[\pi \right]+\sum_{i\in I}\pi^{i}H^{i}\left(\ell \right). \end{array}$$

In the general setting, however, the sequences generated by distinct components can overlap. This reduces the number of distinct positive-probability words and so, too, the block entropy. In this way, we see that the above equality is only an upper bound on the HMSP’s block entropy:

(18) $$\begin{array}{c} H\left(\ell \right)\leq H\left[\pi \right]+\sum_{i\in I}\pi^{i}H^{i}\left(\ell \right). \end{array}$$

This bound highlights the contribution of the *mixture entropy* H[π]. We return to critique this notion of ergodic decomposition later on. For now, we draw out several useful consequences of this line of reasoning, relying on the bound Equation (18). Elsewhere we explore tighter informational bounds on decomposition.

From this, we see that an HMSP’s entropy rate $h_{\mu }$ is simply determined by those of its ergodic components. Assuming the mixture entropy H[π] is finite, we have

(19) $$\begin{array}{cc} h_{\mu } & =\lim_{\ell \to \infty }\frac{H(\ell )}{\ell }\\ \; & \leq \lim_{\ell \to \infty }\frac{1}{\ell }\left\{H\left[\pi \right]+\sum_{i\in I}\pi^{i}H^{i}\left(\ell \right)\right\}\\ \; & =\sum_{i\in I}\pi^{i}\lim_{\ell \to \infty }\frac{H^{i}\left(\ell \right)}{\ell }\\ \; & =\sum_{i\in I}\pi^{i}h_{\mu }^{i}, \end{array}$$

where we have the *component entropy rate* $h_{\mu }^{i}=h_{\mu }\left(M^{i}\right)$. Reference [28] originally established this decomposition.

What is less intuitive, though, are various complexity measures as they apply to HMSPs. As we will see, unlike the entropy rate, which component processes are selected and how they relate to one another play key roles. We first consider the ergodic decomposition for excess entropy, then for the transient information, and finally that for the statistical complexity.

The excess entropy E also has an ergodic decomposition. In this case, we have

(20) $$\begin{array}{cc} E & =\lim_{\ell \to \infty }\left(H\left(\ell \right)-h_{\mu }\ell \right)\\ \; & \leq \lim_{\ell \to \infty }\left(H\left[\pi \right]+\sum_{i\in I}\pi^{i}H^{i}\left(\ell \right)-\ell \sum_{i\in I}\pi^{i}h_{\mu }^{i}\right)\\ \; & =H\left[\pi \right]+\sum_{i\in I}\pi^{i}\left(\lim_{\ell \to \infty }\left[H^{i}\left(\ell \right)-h_{\mu }^{i}\ell \right]\right)\\ \; & =H\left[\pi \right]+\sum_{i\in I}\pi^{i}E^{i}, \end{array}$$

where $E^{i}$ is the excess entropy for ergodic component *i*. The excess entropy decomposition was explored in Refs. [29,30].

Combining the entropy rate and excess entropy ergodic decompositions, we see that the block-entropy linear asymptotes—$H^{i}\left(\ell \right)\propto E^{i}+h_{\mu }^{i}\ell$—have their own decomposition,

(21) $$\begin{array}{cc} E+h_{\mu }\ell & \leq H\left[\pi \right]+\sum_{i\in I}\pi^{i}E^{i}+\ell \cdot \sum_{i\in I}\pi^{i}h_{\mu }^{i}\\ \; & =H\left[\pi \right]+\sum_{i\in I}\pi^{i}\left(E^{i}+h_{\mu }^{i}\ell \right). \end{array}$$

It is a simple additional step to develop the ergodic decomposition for the transient information,

(22) $$\begin{array}{cc} T & =\sum_{\ell =0}^{\infty }\left[E+h_{\mu }\ell -H\left(\ell \right)\right]\\ \; & \leq \sum_{\ell =0}^{\infty }\left[H\left[\pi \right]+\sum_{i\in I}\pi^{i}\left(E^{i}+h_{\mu }^{i}\ell \right)\right.\\ \; & \;\;\;\left.+\ell \sum_{i\in I}\pi^{i}h_{\mu }^{i}-H\left[\pi \right]-\sum_{i\in I}\pi^{i}H^{i}\left(\ell \right)\right]\\ \; & =\sum_{\ell =0}^{\infty }\sum_{i\in I}\pi^{i}\left[E^{i}+h_{\mu }^{i}\ell -H^{i}\left(\ell \right)\right]\\ \; & =\sum_{i\in I}\pi^{i}T^{i}. \end{array}$$

Curiously, like the entropy rate decomposition, the mixture entropy H[π] does not play a role.

The statistical complexity also has an ergodic decomposition,

(23) $$\begin{array}{cc} C_{\mu } & =-\sum_{\sigma \in S}P\left(\sigma \right)\log_{2}P\left(\sigma \right)\\ \; & =-\sum_{i\in I}\sum_{\sigma^{i}\in S^{i}}P\left(\sigma^{i}\right)\log_{2}P\left(\sigma^{i}\right)\\ \; & =-\sum_{i\in I}\sum_{j=0}^{|S^{i}|-1}\pi_{ij}\log_{2}\pi_{ij}\\ \; & =-\sum_{i\in I}\sum_{j=0}^{|S^{i}|-1}\pi^{i}p_{j}^{i}\log_{2}\pi^{i}p_{j}^{i}\\ \; & =-\sum_{i\in I}\pi^{i}\sum_{j=0}^{|S^{i}|-1}p_{j}^{i}\left(\log_{2}\pi^{i}+\log_{2}p_{j}^{i}\right)\\ \; & =-\sum_{i\in I}\pi^{i}\sum_{j=0}^{|S^{i}|-1}p_{j}^{i}\log_{2}\pi^{i}-\sum_{i\in I}\pi^{i}\sum_{j=0}^{|S^{i}|-1}p_{j}^{i}\log_{2}p_{j}^{i}\\ \; & =-\sum_{i\in I}\pi^{i}\log_{2}\pi^{i}-\sum_{i\in I}\pi^{i}C_{\mu }^{i}\\ \; & =H\left[\pi \right]+\sum_{i\in I}\pi^{i}C_{\mu }^{i}, \end{array}$$

where $C_{\mu }^{i}$ are the statistical complexities of the ergodic components. The decomposition for statistical complexity was first noted in Ref. [31]. Note that this decomposition does not rely on assuming an equality as in Equation (18).

Finally, the multistationary crypticity χ, which measures how a process hides state information from an observer, is also unaffected by the mixture distribution,

(24) $$\begin{array}{cc} \chi & =C_{\mu }-E\\ \; & \geq H\left[\pi \right]+\sum_{i\in I}\pi^{i}C_{\mu }^{i}-\left(H\left[\pi \right]+\sum_{i\in I}\pi^{i}E^{i}\right)\\ \; & =\sum_{i\in I}\pi^{i}\left(C_{\mu }^{i}-E^{i}\right)\\ \; & =\sum_{i\in I}\pi^{i}\chi^{i}, \end{array}$$

where $\chi^{i}$ is the crypticity of component $M^{i}$. In this, it is similar to the entropy rate and transient information decompositions.

## 7. Structural Decompositions—Beyond Statistical

To emphasize, what’s notable in these kinds of informational decomposition is that, for nonergodic ϵ-machines, we have, for example,

$$\begin{array}{c} C_{\mu }>\sum_{i\in I}\pi^{i}C_{\mu }^{i}. \end{array}$$

That is, the global structural complexity $C_{\mu }$ of a multistationary process is strictly greater than that contained in its components $\left\{C_{\mu }^{i}\right\}$. In short, a multistationary process is *at least the sum of its parts*. Indeed, the above inequality leaves out the entropy of mixing. But this is too facile. As we will see, multistationary processes are much, much more.

We will see below, taking a more structural perspective going beyond the ergodic decompositions, that the transient causal state structure is key to a process’ global organization and what sequences of observations reveal. This leads us to call into question the interpretation and use of the preceding kinds of ergodic decomposition.

We now show that the construction procedure can be used to answer a number of different questions about multistationary ergodic processes. Several questions are illustrated via particular examples; others via general constructions. The series of examples is developed incrementally to highlight the methods and particular results, as much in isolation as possible.

We first start with processes built from finite-state ergodic components that lead to a multistationary process that is itself finite-state. Then we analyze the case in which finite components lead to a multistationary process with an infinite number of states. We end with examples built from an infinite number of finitary ergodic processes. In each case, we explore the structure of the resulting multistationary process, its complexity measures, and its ergodic decomposition.

### 7.1. Finite Hidden Multistationary Processes

#### 7.1.1. A Base Case

A simple but illustrative case is that of two period-1 component processes: all Heads and all Tails, selected with fair probability: π=(1/2,1/2).

The components observed separately have $h_{\mu }^{0}=h_{\mu }^{1}=0$. But together H(ℓ)=1, ℓ≥1, since that is the informational uncertainty we have about which component the process is in. Naturally, once in a component there is no uncertainty about the symbols emitted. In this way, we see that the HMSP information H(ℓ) of the mixture is all mixing entropy H(π).

The composite ϵ-machine consists of three causal states: a single transient start state that immediately transitions (with fair probability) to either the All-Heads recurrent component (single recurrent state) or to the All-Tails recurrent component (also a single recurrent state).

#### 7.1.2. Period-1 and Period-2 Process

Define the *Periodic Process* P≡P(p) that repeats the word $w=0^{p-1}1$. Let us construct the simplest multistationary process consisting of the following two components:

1. Period-1 process P(1), which has complexity measures $h_{\mu }^{1}=0$ bits per symbol, $C_{\mu }^{1}=0$ bits, $E^{1}=0$ bits, $T^{1}=0$ bit-symbols, and $\chi^{1}=0$ bits.
2. Period-2 process P(2), which has complexity measures $h_{\mu }^{1}=0$ bits per symbol, $C_{\mu }^{1}=1$ bit, $E^{1}=1$ bit, $T^{1}=1$ bit-symbol, and $\chi^{1}=0$ bits.

**Figure 1:**
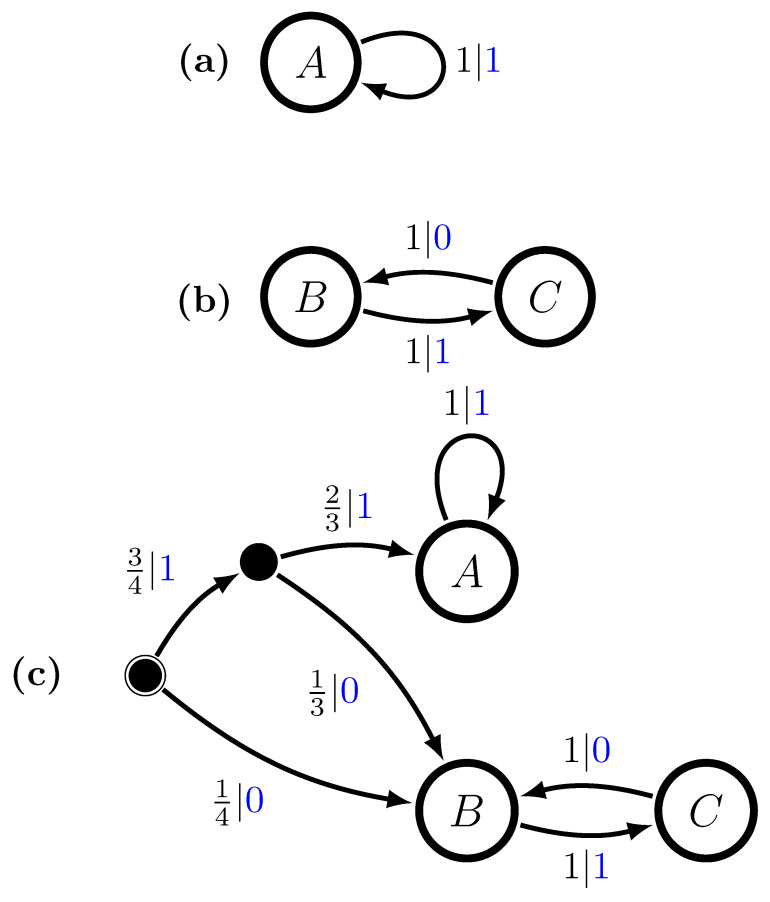
The Period-1 and Period-2 hidden multistationary process: (**a**) Component P(1), (**b**) component P(2), and (**c**) $M=U_{\pi }\left(P\left(1\right)⨆P\left(2\right)\right)$ with $\pi =\left(\frac{1}{2},\frac{1}{2}\right)$. Recurrent causal states are shown as hollow circles and transient causal states as small solid (black) circles. The start state sports a double circle. Transitions are labeled p|x to indicate taking the transition with probability *p* and emitting symbol x∈A.

The Period-1 component has a single recurrent state *A* and the Period-2 has two recurrent states; label them *B* and *C*. The second step is to specify the mixture distribution π and we take this to be uniform: $\pi =\left(\frac{1}{2},\frac{1}{2}\right)$. That is, $P(M^{1})=1/2$ and $P(M^{2})=1/2$. And the final step is to use the mixed-state operator to construct $M=U_{\pi }\left(P(1)⨆P(2)\right)$. The resulting multistationary ϵ-machine is shown in Figure 1c.

The recurrent states of the component ϵ-machines show up as *M*’s recurrent states, as claimed. The two recurrent state sets are not connected. What is new are the two transient states (solid circles). As a generator of the multistationary process, *M* begins in its start state (solid circle with circumscribing circle) and then follows transitions according to the edge probabilities, emitting the corresponding symbols, eventually reaching one or the other of the two recurrent-state sets—{A} or {B,C}.

We can understand *M*’s structure by calculating its mixed states $\mu \left(w\right)=\left(P(A),P(B),\right.$$\left.P(C)\right)$, w∈A, using Equation (9):

$$\begin{array}{cc} \mu (\lambda ) & =\left[\frac{1}{2},\frac{1}{4},\frac{1}{4}\right],\\ \mu (0) & =\left[0,1,0\right]=B,\\ \mu (1) & =\left[\frac{1}{2},0,\frac{1}{2}\right],\\ \mu (00) & =\left[0,0,0\right]=\emptyset ,\\ \mu (01) & =\left[0,0,1\right]=C,\\ \mu (10) & =\left[0,1,0\right]=B,\;\mathrm{and}\\ \mu (11) & =\left[1,0,0\right]=A. \end{array}$$

In this, on the one hand, μ(λ) is the start state of the mixed state presentation and its distribution gives the asymptotic invariant distribution over the component recurrent states *A*, *B*, and *C*—the state probabilities before any symbols have been generated.

On the other hand, if x=0 is generated, then we immediately know the process is in component P(2), since P(1) cannot produce a 0, and, in particular, it is in a specific state, *B*. This is reflected in the transient mixed state $\mu \left(0\right)=\left[0,1,0\right]$. In fact, any time a valid 0 is generated, we know *M* is in state *B*. This is also seen in the mixed state μ(10), in which the last symbol generated is a 0 and we again obtain a δ-function distribution concentrated on state *B*.

Now, there are also disallowed transitions and so disallowed words. This is shown in the mixed state $\mu \left(00\right)=\left[0,0,0\right]$ for the word w=00.

More interesting, though, is the transient mixed state $\mu \left(1\right)=\left[\frac{1}{2},0,\frac{1}{2}\right]$, which indicates that, having seen a 1, we know that *M* cannot be in state *B*. However, the best we can say is that it is either in state *A* (the Period-1 component) or in state *C* (the Period-2 component) with fair probability. It is not until we see another symbol that we are guaranteed to know with certainty in which component *M* is. If w=11, then P is in *A*. Since we now know the state with certainty, we say that w=0 and w=11 are *synchronizing words*. In this case, they are the minimal synchronizing words.

The ergodic decompositions tell us the following:

1. $h_{\mu }=\pi^{1}h_{\mu }^{1}+\pi^{1}h_{\mu }^{2}=0$ bit per symbol;
2. $E=H\left(\pi \right)+\pi^{1}E^{1}+\pi^{2}E^{2}=1+0+1/2=1.5$ bits;
3. $C_{\mu }=H\left(\pi \right)+\pi^{1}C_{\mu }^{1}+\pi^{2}C_{\mu }^{2}=1+0+1/2=1.5$ bits;
4. $T=\pi^{1}T^{1}+\pi^{1}T^{2}=0+1/2=1/2$ bit-symbols;
5. $\chi =\pi^{1}\chi^{1}+\pi^{1}\chi^{2}=0+0=0$ bits.

**Figure 2:**
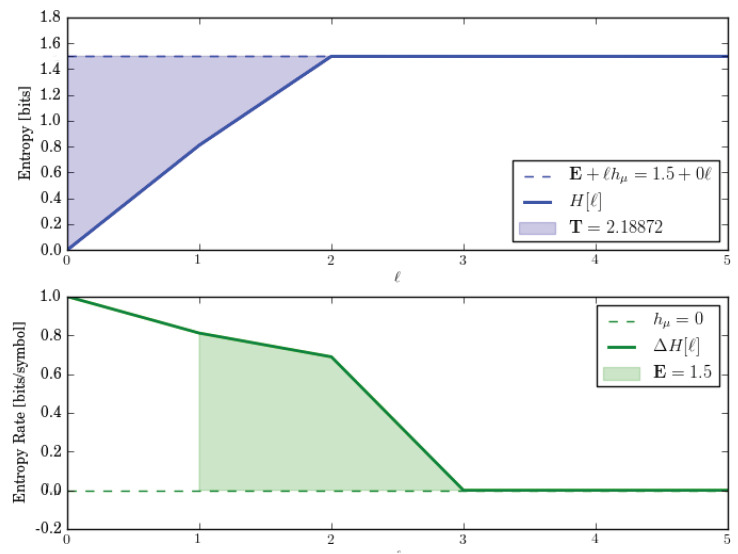
Entropy growth H(ℓ) (**top**) and entropy convergence $h_{\mu }\left(\ell \right)$ (**bottom**) for the Period-1 and Period-2 HMSP, as function of word length ℓ=0,…,5.

Let us check these by directly calculating the entropy growth H(ℓ) and convergence $h_{\mu }\left(\ell \right)$ for *M*. These are shown in Figure 2.

The entropy growth plot (top) leads to an estimate of E≈1.5 bits, which is predicted by the ergodic decomposition. Both entropy growth and entropy convergence (bottom) show that $h_{\mu }\left(\ell \right)=h_{\mu }=0$ after ℓ=2. And this too is correctly predicted by the corresponding entropy rate decomposition.

In fact, for lengths longer than the longest period, there are always three distinct sequences—w∈{1111…,0101…,1010…}. And so, $E\leq \log_{2}3\approx 1.585$ bits. This is roughly consistent with block entropy plots.

Let us analyze this exactly. One of those sequences is $w=1^{n}$ and it occurs with probability 1/2. The two other sequences are $w={\left(01\right)}^{n}$ and $w={\left(10\right)}^{n}$ and they are generated equally often by their components. But since that component appears only half the time, they occur in the output sequences with probability 1/4 each. Thus, E=H[P(w)]=H[(1/2,1/4,1/4)]=1.5 bits. And this is what is seen in the plots.

The HMSP’s statistical complexity is

(25) $$\begin{array}{cc} C_{\mu } & =H\left[P(S)\right]\\ \; & =H\left[(1/2,1/4,1/4)\right]\\ \; & =3/2\;\mathrm{bits}. \end{array}$$

which agrees with the ergodic decomposition.

The ergodic decomposition, however, predicts T=1/2 bit-symbols, while the entropy growth plot shows that, in fact, T≈2.19 bit-symbols. So, the ergodic decomposition for T is incorrect. In short, we see that the ergodic decomposition does not properly account for the state distribution’s relaxation through the transient mixed states (solid circles) in *M*; Figure 1c. That relaxation takes longer than a single step (as the decomposition incorrectly assumes) and that increased relaxation time increases T.

Note that this is one of the simpler examples of the class of processes that have finite transients. Let us consider one that is more complex.

#### 7.1.3. Isomorphic Golden Means Process

The No-Repeated-0s Golden Mean Process (GMP) generates all binary sequences except those with consecutive 0s. When a 0 is generated, then the probability of a 0 or a 1 is fair. The GMP is an order-1 Markov process.

**Figure 3:**
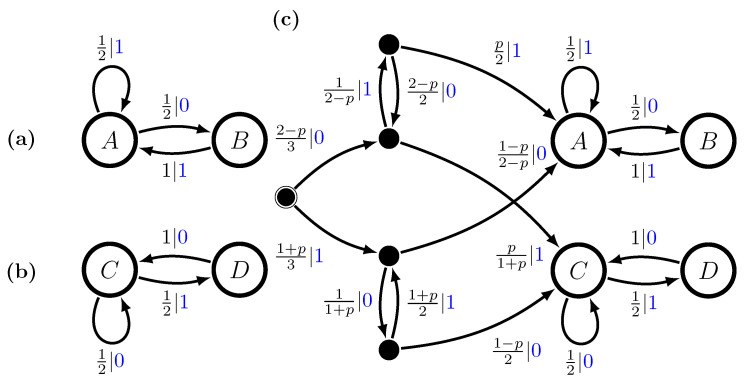
Two Golden Mean Processes and their nonergodic mixture: (**a**) $M^{1}$, (**b**) $M^{2}$, and (**c**) $M=U_{\pi }\left(M^{1}⨆M^{2}\right)$ with π=(p,1−p).

Let $P^{1}$ be the No-Repeated-0s GMP, and let $P^{2}$ be the No-Repeated-1s GMP. See Figure 3a,b. We define a nonergodic mixture P as follows:

$$\begin{array}{c} P=p\;P^{1}+\left(1-p\right)\;P^{2}, \end{array}$$

with mixture distribution π=(p,1−p). The probability of any word *w* is, then,

$$\begin{array}{c} P\left(w\right)=p\;P_{1}\left(w\right)+\left(1-p\right)\;P_{2}\left(w\right). \end{array}$$

Using the mixed-state operator, we construct M(P)’s transient and recurrent states using this mixture distribution, finding

$$\begin{array}{cc} \mu (w) & =\left[\begin{array}{cccc} P(A|w) & P(B|w) & P(C|w) & P(D|w) \end{array}\right],\\ \mu (\lambda ) & =\left[\begin{array}{cccc} \frac{2p}{3} & \frac{p}{3} & \frac{2(1-p)}{3} & \frac{1-p}{3} \end{array}\right]\;\\ \mu (0) & =\left[\begin{array}{cccc} 0 & \frac{p}{2-p} & \frac{2(1-p)}{2-p} & 0 \end{array}\right],\\ \mu (1) & =\left[\begin{array}{cccc} \frac{2p}{1+p} & 0 & 0 & \frac{1-p}{1+p} \end{array}\right],\\ \mu (00) & =\left[\begin{array}{cccc} 0 & 0 & 1 & 0 \end{array}\right]=C,\\ \mu (01) & =\left[\begin{array}{cccc} p & 0 & 0 & 1-p \end{array}\right],\\ \mu (10) & =\left[\begin{array}{cccc} 0 & p & 1-p & 0 \end{array}\right],\\ \mu (11) & =\left[\begin{array}{cccc} 1 & 0 & 0 & 0 \end{array}\right]=A,\\ \mu (001) & =\left[\begin{array}{cccc} 0 & 0 & 0 & 1 \end{array}\right]=D,\;\mathrm{and}\\ \mu (110) & =\left[\begin{array}{cccc} 0 & 1 & 0 & 0 \end{array}\right]=B. \end{array}$$

Longer words can only lead to one of these mixed states and so the ϵ-machine is finite. The full multistationary ϵ-machine is shown in Figure 3c, as a function of the mixture parameter *p*. We see that the number of states, including the transients, is finite for all mixture probabilities.

The transition matrices for M(P)’s recurrent causal states are

$$T^{0}=\left[\begin{array}{cccc} 0 & \frac{1}{2} & 0 & 0\\ 0 & 0 & 0 & 0\\ 0 & 0 & \frac{1}{2} & 0\\ 0 & 0 & 1 & 0 \end{array}\right]\;\mathrm{and}\;T^{1}=\left[\begin{array}{cccc} \frac{1}{2} & 0 & 0 & 0\\ 1 & 0 & 0 & 0\\ 0 & 0 & 0 & \frac{1}{2}\\ 0 & 0 & 0 & 0 \end{array}\right].$$

The stationary distribution is defined by the mixture of the two processes,

$$\begin{array}{cc} \pi (p) & =\left(\begin{array}{cc} p\pi^{1} & \left(1-p\right)\pi^{2} \end{array}\right)\\ \; & =\frac{1}{3}\left(\begin{array}{cccc} 2p & p & 2(1-p) & 1-p \end{array}\right), \end{array}$$

recalling that $\pi^{1}=\pi^{2}=\left(\begin{array}{cc} 2/3 & 1/3 \end{array}\right)$.

Using methods from refs. [16,17], the excess entropy for each recurrent component is seen to be

$$\begin{array}{cc} E^{1}=E^{2} & =\frac{2}{3}\log_{2}\frac{3}{2}+\frac{1}{3}\log_{2}3-\frac{2}{3}\\ \; & =\frac{2}{3}\log_{2}\frac{3}{4}+\frac{1}{3}\log_{2}3\\ \; & \approx 0.251629\;\mathrm{bits}. \end{array}$$

By the ergodic decomposition theorem, the excess entropy for the mixture, as a function of *p* is

$$\begin{array}{cc} E(p) & =pE^{1}+\left(1-p\right)E^{2}+H\left(p\right)\\ \; & =E^{1}+H\left(p\right), \end{array}$$

since the two components are isomorphic. For p=1/2, we expect E≈1.251629 bits.

Again, the component transient information equals the excess entropy, since the GMP is order-1 Markov. So, the associated ergodic decomposition gives

$$\begin{array}{cc} T(p) & =pT^{1}+\left(1-p\right)T^{2}\\ \; & =T^{1}, \end{array}$$

since the two components are isomorphic. For p=1/2, we expect T≈0.251629 bits.

Similarly, the statistical complexity of each recurrent component is

$$\begin{array}{cc} C_{\mu }^{1}=C_{\mu }^{2} & =\frac{2}{3}\log_{2}\frac{3}{2}+\frac{1}{3}\log_{2}3\\ \; & \approx 0.9182958\;\mathrm{bits}. \end{array}$$

So, from Equation (23) the statistical complexity of the mixture as a function of *p* is

(26) $$\begin{array}{c} C_{\mu }\left(p\right)=C_{\mu }^{1}+H\left(p\right). \end{array}$$

For p=1/2, we expect $C_{\mu }\approx 1.9182958$ bits.

**Figure 4:**
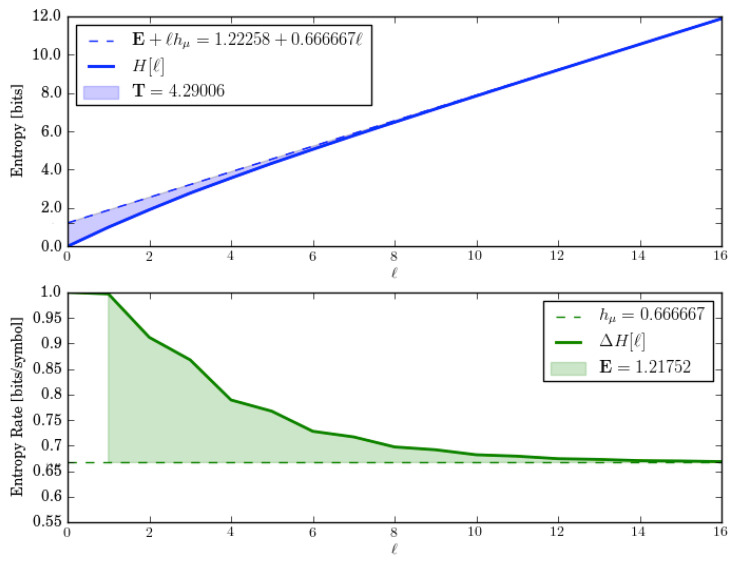
Entropy growth H(ℓ) (**top**) and entropy convergence $h_{\mu }\left(\ell \right)$ (**bottom**) for the Two Isomorphic Golden Means HMSP, as function of word length ℓ=0,…,16 and mixture parameter p=1/2.

Let us check the decompositions by calculating the associated complexity measures from *M*’s entropy growth and convergence. The latter are shown in Figure 4.

The entropy growth plot estimates that E=1.22258 bits, which is low by 2%. And the entropy convergence plot shows that the E, calculated there using Equation (5) as the area shown, is a bit lower still: E=1.21753. Although, due to the slow convergence and the finite number of terms taken in the approximation, these errors are expected. Similarly, the ergodic decomposition of the entropy rate $h_{\mu }=0.666667$ bits per symbol shows up correctly when estimated from P’s entropy growth and convergence. And so, the predictions from the related ergodic decompositions are consistent.

The entropy growth, however, shows the transient information is substantially larger (T≈4.29 bit-symbols) than that predicted from its ergodic decomposition (T=0.251629). This discrepancy is clearly not due to estimation errors. Rather, as noted above for the P1-P2 mixture, it arises from the decomposition not accounting for the five transient causal states of *M*; see Figure 3c.

Individually, GMPs are subshifts of finite type and finite Markov order. From the cycles in the transient states, we see that as components they make the multistationary process sofic—infinite Markov order. There are subsets of sequences—specifically ${\left(01\right)}^{n}$—for which one never synchronizes.

This means that mixtures of finite-order Markov chains, even “linear” mixtures that come from independently running them, are processes that are not finite Markovian. They require hidden Markov representations.

### 7.2. Infinite State

The preceding examples, chosen to explicitly illustrate methods and as harbingers of coming results, are rather special in that the led to finite-state multistationary processes. We now turn to more typical cases, still constructed from finite-state ergodic components, that lead to a multistationary process with an infinite number of states.

#### 7.2.1. Period-1 and Fair Coin Process

The next example of a multistationary process mixes stochastic and periodic behaviors: We build it out of a period-1 process and a fair coin. In effect, we ask how difficult it is to distinguish these two simple, but extreme processes—one completely predictable, the other completely unpredictable.

For here and a bit later, define *Bernoulli Process* $B\left(p\right)$, which is a model of a coin flip with bias probability *p*.

**Figure 5:**
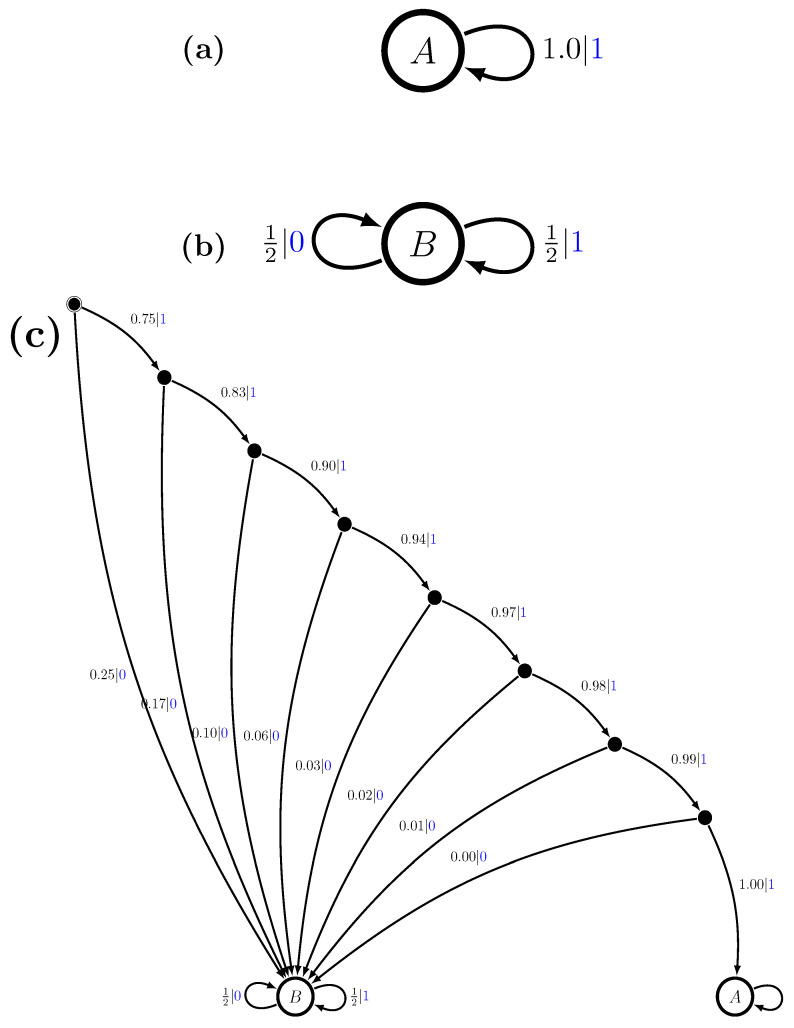
Period-1 and Fair Coin HMSP: (**a**) P(1), (**b**) $B\left(\frac{1}{2}\right)$, and (**c**) $M=U_{\pi }\left(P\left(1\right)⨆B\left(\frac{1}{2}\right)\right)$ with $\pi =\left(\frac{1}{2},\frac{1}{2}\right)$. The latter is approximated by connecting the Period-1 component after 8 1s. In fact, the P(1) component is never reached after any finite sequence. In contrast, $B\left(\frac{1}{2}\right)$ can be reached quickly and by many sequences. Recurrent causal states are shown as hollow circles and transient causal states as small solid (black) circles. The start state sports a double circle. Transitions are labeled p|x to indicate taking the transition with probability *p* and emitting symbol x∈A.

The first step, then, is to select the following two stationary components:

1. Period-1 Process P(1): $h_{\mu }^{1}=0$ bits per symbol, $C_{\mu }^{1}=0$ bits, $E^{1}=0$ bits, $T^{1}=0$ bit-symbols, and $\chi^{1}=0$ bits. See Figure 5a.
2. Fair Coin Process $B\left(\frac{1}{2}\right)$:, $h_{\mu }^{1}=1$ bit per symbol, $C_{\mu }^{1}=0$ bits, $E^{1}=0$ bits, $T^{1}=0$ bit-symbols, and $\chi^{1}=0$ bits. See Figure 5b.

Though at the two extremes of predictability, these are structurally trivial processes—$C_{\mu }^{i}=0$.

The second step is to select the mixture distribution, which we take to be uniform: $\pi =\left(\frac{1}{2},\frac{1}{2}\right)$. And the third step is to use the mixed-state operator to construct $M=U_{\pi }\left(P\left(1\right)⨆B\left(\frac{1}{2}\right)\right)$. Several of the mixed states are

$$\begin{array}{cc} \mu (\lambda ) & =\left[\frac{1}{2},\frac{1}{2}\right],\\ \mu (0) & =\left[0,1\right]=B\\ \mu (1) & =\left[\frac{3}{4},\frac{1}{4}\right],\\ \mu (0A^{+}) & =\left[0,1\right]=B,\\ \mu (1^{+}0) & =\left[0,1\right]=B,\\ \mu (1^{k}) & =\left[1-\alpha_{k},\alpha_{k}\right],\\ \; & \vdots ,\;\mathrm{and}\\ \mu (1^{\infty }) & =\left[1,0\right]=A, \end{array}$$

where $\alpha_{k}=\left(2k+1\right)/2^{k+1}$. The resulting ϵ-machine is shown in Figure 5c.

In Figure 5c, and so too in the mixed states, we see our first surprising result for multistationary processes. Starting from two structurally trivial processes, the multistationary ϵ-machine has a countable infinity of transient causal states. Why? If, at any point, one sees a 0, then we know the process is in the Fair Coin component, since the other component cannot generate a 0. However, it is only after “seeing” an infinite sequence of 1s that one could determine that the process is in the All-1s component. In short, the effort required to distinguish between these two trivial processes is infinite and this is directly reflected in the infinite set of transient states.

The ergodic decompositions tell us the following:

1. $h_{\mu }=\pi^{1}h_{\mu }^{1}+\pi^{2}h_{\mu }^{2}=0+1/2=1/2$ bit per symbol;
2. $E=H\left(\pi \right)+\pi^{1}E^{1}+\pi^{2}E^{2}=1+0+0=1$ bits;
3. $C_{\mu }=H\left(\pi \right)+\pi^{1}C_{\mu }^{1}+\pi^{2}C_{\mu }^{2}=1+0+0=1$ bits;
4. $T=\pi^{1}T^{1}+\pi^{2}T^{2}=0+0=0$ bit-symbols;
5. $\chi =\pi^{1}\chi^{1}+\pi^{2}\chi^{2}=0+0=0$ bits.

**Figure 6:**
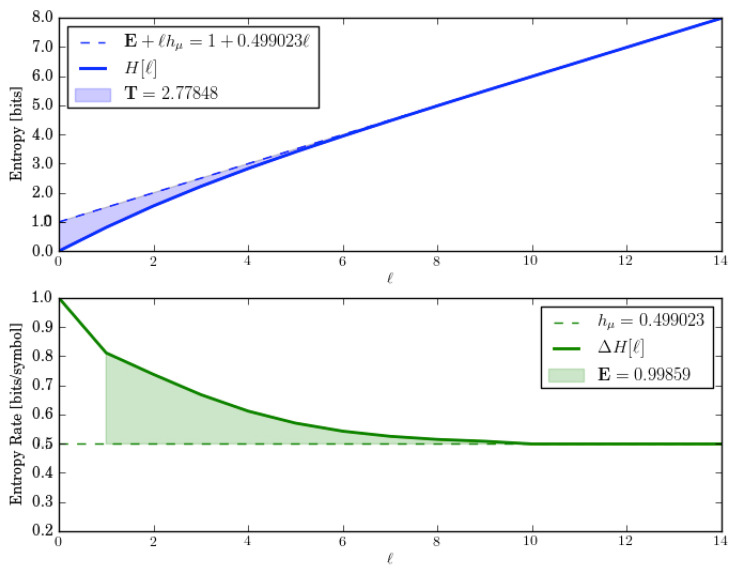
Entropy growth H(ℓ) (**top**) and entropy convergence $h_{\mu }\left(\ell \right)$ (**bottom**) for the Period-1 and Fair Coin HMSP, as function of word length ℓ=0,…,14.

Note that the ergodic decompositions predict that the structural complexity measures are driven solely by the mixture entropy H(π). Both components contribute nothing: $E^{i}=C_{\mu }^{i}=0$. Let us check these predictions by estimating the quantities from *M*’s entropy growth and convergence, shown in Figure 6.

The entropy growth plot shows that E=1 bit, as predicted by *M*’s ergodic decomposition. And the entropy convergence plot shows that the E, calculated as the area shown, is also the same. Similarly, the ergodic decomposition of the entropy rate $h_{\mu }=\frac{1}{2}$ bits per symbol shows up correctly on the entropy plots.

The entropy growth plot, however, shows the transient information is quite a bit larger (T≈2.78 bit-symbols) than that predicted (T=0) from its ergodic decomposition.

Also, the informational ergodic decompositions, while indicating a role for the mixture entropy, miss entirely the existence of an infinite number of transient states and the attendant difficulty that confronts an observer trying to detect in which component the process is.

#### 7.2.2. Two Biased Coins

**Figure 7:**
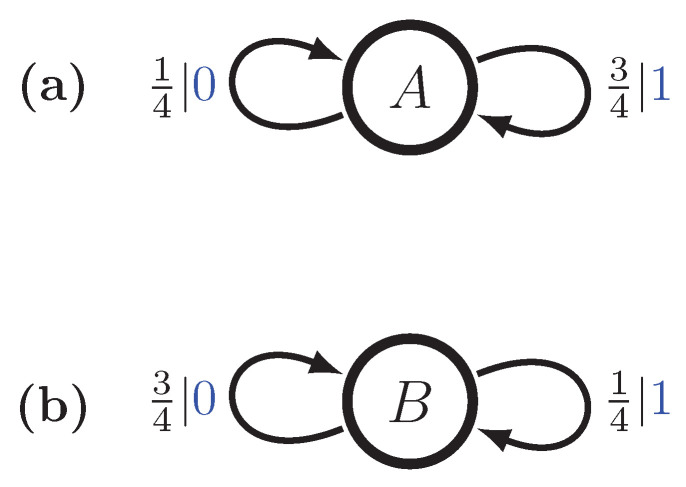
Two Biased Coins HMSP Components: (**a**) $B\left(\frac{1}{4}\right)$ and (**b**) $B\left(\frac{3}{4}\right)$.

Slightly increasing the level of sophistication, we now construct a multistationary process out of fully stochastic components: Two biased coins of unequal (but symmetric) biases $B\left(\frac{1}{4}\right)$ and $B\left(\frac{3}{4}\right)$. See Figure 7a,b.

**Figure 8:**
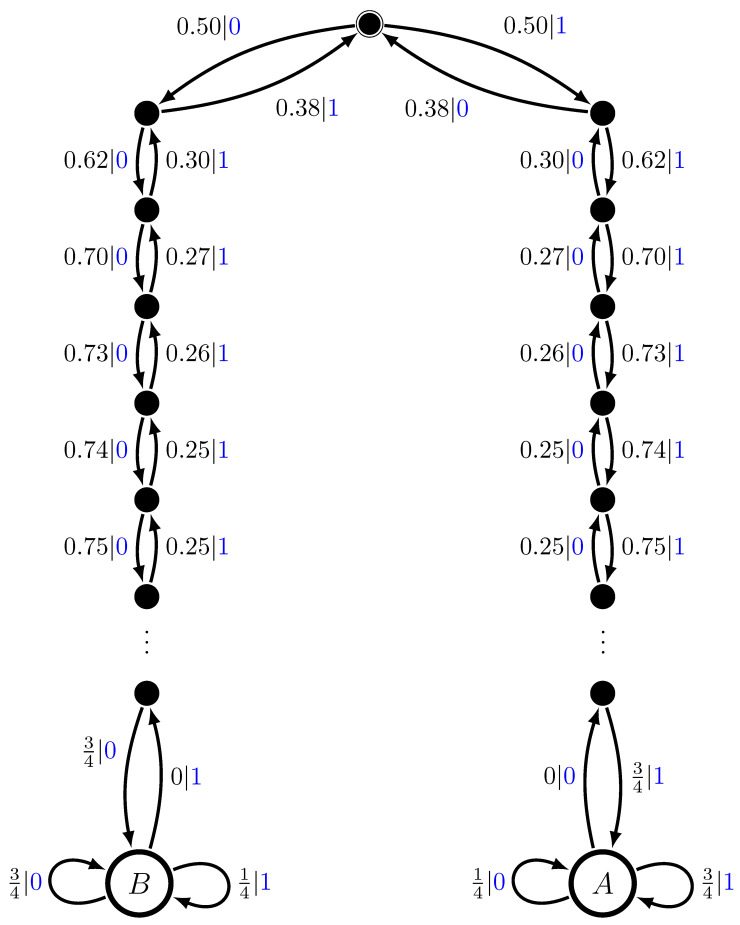
Two Biased Coins HMSP: $M=U_{\pi }\left(B\left(\frac{1}{4}\right)⨆B\left(\frac{3}{4}\right)\right)$ with $\pi =\left(\frac{1}{2},\frac{1}{2}\right)$. The latter is approximated by connecting the transient states to $B\left(\frac{1}{4}\right)$ and $B\left(\frac{3}{4}\right)$ after 30 0s or 30 1s, respectively. In fact, $B\left(\frac{1}{4}\right)$ and $B\left(\frac{3}{4}\right)$ are never reached after any finite word.

We again take a uniform mixture distribution: $\pi =\left(\frac{1}{2},\frac{1}{2}\right)$. The result of constructing $M=U_{\pi }\left(B\left(\frac{1}{4}\right)⨆B\left(\frac{3}{4}\right)\right)$ with $\pi =\left(\frac{1}{2},\frac{1}{2}\right)$ is shown in Figure 8.

The mixed state presentation reveals two countably and infinitely long chains of transient causal states. One leads to the ergodic component for $B\left(\frac{3}{4}\right)$ and the other for $B\left(\frac{1}{4}\right)$. In a simple sense these long transient chains show the mechanism by which one determines the coin biases. Interestingly, though, at any point statistical fluctuations can change the apparent bias and drive the state back up the long chains, heading for the complementary biased coin.

Consider, as above the $h_{\mu }$, E, $C_{\mu }$, T, and χ ergodic decompositions. The ergodic decompositions for excess entropy and statistical complexity give similar results; namely,

$$\begin{array}{cc} E & =H\left[\pi \right]+E\left(M^{1}\right)+E\left(M^{2}\right)\\ E & =H[\pi ],\;\mathrm{and}\\ C_{\mu } & =H\left[\pi \right]+C_{\mu }\left(M^{1}\right)+C_{\mu }\left(M^{2}\right)\\ C_{\mu } & =H[\pi ]. \end{array}$$

That is, the complexities of the multistationary process are all in the mixture distribution. Even then, the mixture entropy, in this case a number upper bounded by 1, belies the infinite number of transients and the difficulty of determining in which ergodic component the process is. Quantitatively, it seems another measure of the global process complexity and a new decomposition are in order. We return to this shortly, after examining several more kinds of multistationary processes.

**Figure 9:**
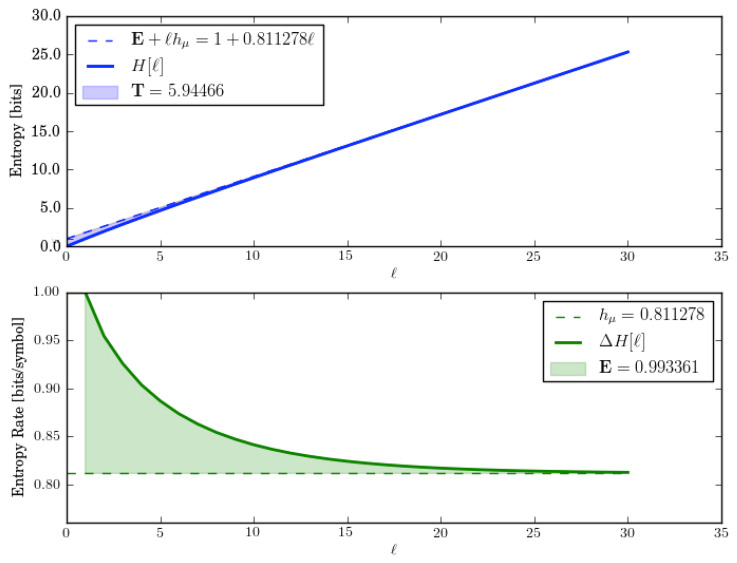
Entropy growth H(ℓ) (**top**) and entropy convergence $h_{\mu }\left(\ell \right)$ (**bottom**) for the Two Biased Coins HMSP, as function of word length ℓ=0,…,30.

Let us validate the ergodic decompositions’ predictions vis-à-vis the process estimates of their various measures from *M*’s entropy growth and convergence. The latter are shown in Figure 9.

Entropy growth, using the *y*-intercept method, shows that E=1 bit, as predicted by *M*’s ergodic decomposition. And the entropy convergence plot shows that E, as the area shown, is also the same, though it takes many terms and so shows slow convergence. The ergodic decomposition of the entropy rate $h_{\mu }=0.811278$ bits per symbol shows up correctly on the entropy plots.

The entropy growth, however, shows the transient information is substantially larger (T≈5.95 bit-symbols) than that predicted (T=0) from its ergodic decomposition. Again, the mixing entropy fails to account for the dominating transient causal state structure.

#### 7.2.3. Pair of Isomorphic Even Processes

The Even Process (EP) generates all binary sequences such that pairs of 1s occur in blocks of even length bounded by 0s. Once a 0 is seen, a 0 or a 1 is generated with fair probability. The EP is closely related to the Golden Mean Process. They have the same entropy rates and statistical complexities. The main important difference, despite the close similarity and a simple relabeling of transitions, is that the EP is described by no finite-order Markov chain. It is infinite Markov order, though finite state.

**Figure 10:**
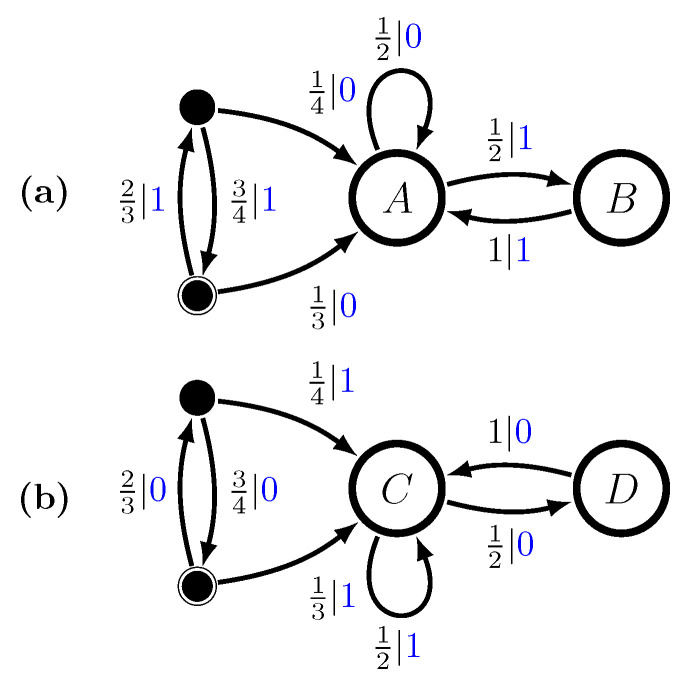
Two Even Processes: (**a**) $M^{1}$, pairs of 1s, with its two transient causal states and (**b**) $M^{2}$, pairs of 0s, with its two transient causal states.

To construct a multistationary process the first step, then, is to select two stationary components. One component $P^{1}$ will be an EP with an even number of 0s (Figure 10b) and the other $P^{2}$ an EP with an even number of 1s (Figure 10a). The second step is to choose mixture distribution: $\pi \left(M^{1},M^{2}\right)=\left(1/2,1/2\right)$.

**Figure 11:**
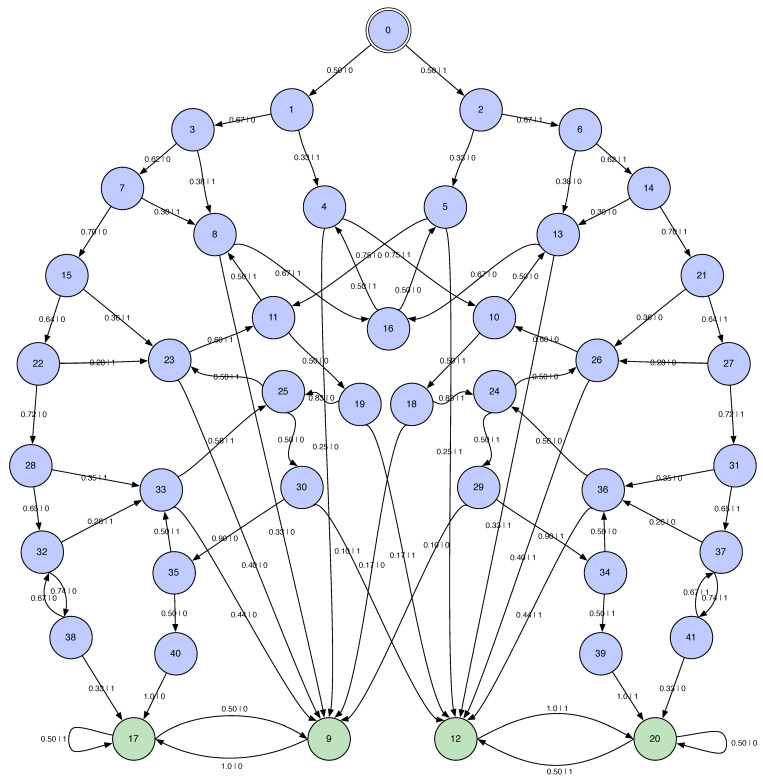
Two Isomorphic Even Process HMSP: $M=U_{\pi }\left(M^{1}⨆M^{2}\right)$ with $\pi \left(M^{1},M^{2}\right)=\left(\frac{1}{2},\frac{1}{2}\right)$. Approximated with maximum word length of 8. Transient states are reconnected to mimic the component EP’s transient states. Specifically, calculating *M* using word length 8 leaves *M*’s states 38−41 as dangling states—with no outgoing transitions. Transitions were added by noting that these states closely approximate the EPs’ two transient states, seen in Figure 10a,b, at sufficiently large word length.

Finally, Figure 11 shows the ϵ-machine for the HMSP $M=U_{\pi }\left(M^{1}⨆M^{2}\right)$ with $\pi \left(M^{1},M^{2}\right)=\left(\frac{1}{2},\frac{1}{2}\right)$. The ϵ-machine displayed is estimated only up to words of length 8 and the transitions are set to give a well-formed ϵ-machine at this approximation.

There are several observations. First, the HMSP ϵ-machine is symmetric under 1-0 exchange, as it should be given this symmetry in the ergodic components. Second, and less obviously, there is an infinite number of transient causal states. This is due to the outside paths along $1^{\infty }$ and $0^{\infty }$. These two sequences arise from the 2-cycles in the respective ergodic component recurrent states: pairs of 1s in $M^{1}$ never synchronize; ditto for pairs of 0s in $M^{2}$. And so, in *M* there are infinitely long sequences that never reach $M^{1}$ or $M^{2}$.

Third, the HMSP is infinite Markov order. To see this, note that there are six cycles in the transient states—these cycles are the signature of infinite Markov order or, what is called “soficity”. The HMSP is a shift in infinite type [32]. In particular, there is a two-cycle ${\left(00\right)}^{+}$ between states 32 and 38 and one ${\left(11\right)}^{+}$ between states 37 and 41. There are two four-cycles ${\left(1100\right)}^{+}$ between states, 10, 18, 24, and 26 and between states 24, 29, 34, and 36; and two ${\left(0011\right)}^{+}$ between states 11, 19, 25, and 23 and between states 25, 30, 35, and 33.

The ergodic decompositions tell us the following:

1. $h_{\mu }=\pi^{1}h_{\mu }^{1}+\pi^{1}h_{\mu }^{2}=h_{\mu }^{1}=\frac{2}{3}$ bit per symbol;
2. $E=H\left(\pi \right)+\pi^{1}E^{1}+\pi^{2}E^{2}=H\left(\pi \right)+E^{1}=1+0.918296=1.918296$ bits;
3. $C_{\mu }=H\left(\pi \right)+\pi^{1}C_{\mu }^{1}+\pi^{2}C_{\mu }^{2}=H\left(\pi \right)+C_{\mu }^{1}=1+0.918296=1.918296$ bits;
4. $T=\pi^{1}T^{1}+\pi^{1}T^{2}=T^{1}=3.16938$ bit-symbols;
5. $\chi =\pi^{1}\chi^{1}+\pi^{1}\chi^{2}=\chi^{1}=0$ bits.

**Figure 12:**
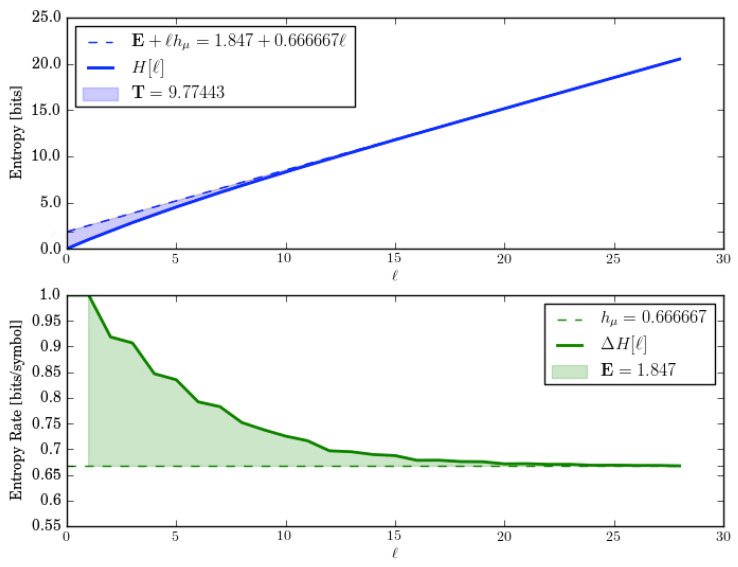
Entropy growth H(ℓ) (**top**) and entropy convergence $h_{\mu }\left(\ell \right)$ (**bottom**) for the Two Isomorphic Even Multistationary Process, as function of word length ℓ=0,…,28.

Let us check these by directly calculating the entropy growth and convergence for *M*. These are shown in Figure 12.

Let us check the decompositions by comparing their predictions to estimates from *M*’s entropy growth and convergence. These functions are shown in Figure 12.

The entropy growth plot shows that E=1.847 bits which disagrees by about 4% with the prediction from the ergodic decomposition (E=1.918 bits). And the entropy convergence plot shows that the E=1.847 bits as the area shown. Although, due to the slow convergence, the finite number of terms taken in the numerical approximation, and the finite number of transient states taken in the approximation of *M*, this error is not surprising. Similarly, the ergodic decomposition of the entropy rate $h_{\mu }=2/3$ bits per symbol shows up correctly on the entropy plots ($h_{\mu }\approx =0.6666$).

Entropy growth, however, shows the transient information is three times larger (T≈9.77443 bit-symbols) than that predicted from its ergodic decomposition (T=3.16938 bit-symbols). Again, this discrepancy follows from the mixture entropy missing the contributions from the (infinite) number of transient causal states.

### 7.3. Infinite Components

We end our selection of example multistationary processes by constructing several from an infinite number of finitary ergodic components.

#### 7.3.1. Handbag of Necklaces

**Figure 13:**
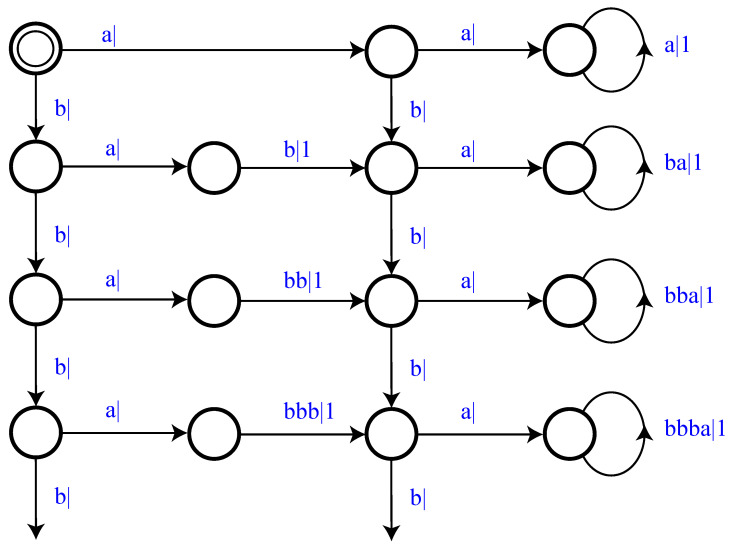
The ϵ-machine for HMSP presentation of the Handbag of Necklaces Process. Chains of probability-1 transitions between causal states are given as strings.

Fourier analysis of a signal assumes the generating process consists of (at most) periodic sequences. As an analog of this assumption in the present setting, consider the *Handbag of Necklaces* (HMSP) consisting of ergodic-component stationary processes P(i) for all periods, i∈I=1,2,3,4,…. That is, if we assume a binary process, the sequences emitted consist of words period-1 $a^{+}$, period-2 ${\left(ab\right)}^{+}$, period-3 ${\left(bba\right)}^{+}$, period-4 ${\left(bbba\right)}^{+}$, and so on. The HMSP ϵ-machine is shown in Figure 13.

Note that there is an infinite number of transient causal states. Overall the HMSP is a highly symmetric structure and dominated by the transient states. From this one can readily read off how to synchronize—how to know in which ergodic component the process is. For example, to obtain to component $M^{i}$, there are exactly *i* paths.

Now, consider the mixture measure $\pi^{i}$ for the components. Then, the state probabilities are $\pi_{ij}=K\pi^{i}/j,\;j=1,\ldots ,i$, where $K=\sum_{i=1}^{\infty }\sum_{j=1}^{i}\pi^{i}/j$ is the normalization constant. Note this is the presentations’ stationary invariant distribution.

There is some flexibility in setting the mixture distribution $\pi^{i}$. There are several criteria for choosing it for a countable number of states, including the following:

1. Normalization: $\sum_{i=1}^{\infty }\sum_{j=1}^{i}\pi_{ij}=\sum_{i=1}^{\infty }\pi^{i}=1$. And so, $\pi^{i}$ must decay faster than 1/i.
2. Finitary (E<∞) HMSP [19]: Must have $H\left[\pi \right]=\sum_{i=1}^{\infty }\pi^{i}\log_{2}\pi^{i}<\infty$.
3. Infinitary (E→∞) HMSP [19]: Must have H[π]→∞. See Ref. [33] for another example process in this family.

Consider the structure of the transitions in the HMSP’s first row of states. The first transition probability for seeing an *a* is

$$\begin{array}{cc} P(a) & =\sum_{i=1}^{\infty }P\left(a|M^{i}\right)P\left(M^{i}\right)\\ \; & =\sum_{i=1}^{\infty }\pi^{i}/i, \end{array}$$

since the probability of seeing an *a* in the $i^{th}$ component is 1/i. Let $p_{a}=P\left(a\right)$. The probability for the succeeding transition emitting an *a* is

$$\begin{array}{cc} P(a|a) & =P(aa)/P(a)\\ \; & =\pi^{1}/p_{a}, \end{array}$$

since $P\left(aa\right)=\pi^{1}$. These transitions determine those leaving the top row of states on a *b*. Note that $P(a^{n}|aa)=1,n=1,2,\ldots$.

Now, consider the second to the top row of transitions. First, $P\left(b\right)=1-p_{a}$. Then, we have

$$\begin{array}{cc} P(a|b) & =P(ba)/P(b)\\ \; & =\frac{p_{a}-\pi^{1}}{1-p_{a}}. \end{array}$$

since

$$\begin{array}{cc} P(ba) & =\sum_{i=2}^{\infty }P\left(M^{i}\right)P\left(ba|M^{i}\right)\\ \; & =\sum_{i=2}^{\infty }\pi^{i}/i\\ \; & =p_{a}-\pi^{1}. \end{array}$$

Note that P(bab)=P(ba). There is a second path to $M^{2}$ controlled by the transition

$$\begin{array}{cc} P(b|a) & =P(ab)/P(a)\\ \; & =\frac{p_{a}-\pi^{1}}{p_{a}}. \end{array}$$

since P(ab)=P(ab). That is, the appearance of ab and ba in each component occurs due to the same conditions.

The ergodic decompositions tell us the following:

1. $h_{\mu }=\sum_{i=1}^{\infty }\pi^{i}h_{\mu }^{i}=0$, as $h_{\mu }\left[P\left(i\right)\right]=0$.
2. The excess-entropy ergodic decomposition for a process with *p* component periodic processes with periods 1,…,p is
  (27) $$\begin{array}{cc} E & =H\left[\pi \right]+\sum_{i=1}^{p}\pi^{i}E^{i} \end{array}$$
  (28) $$\begin{array}{cc} \; & =H\left[\pi \right]+\sum_{i=1}^{p}\pi^{i}\log_{2}i \end{array}$$
  (29) $$\begin{array}{cc} \; & =\log_{2}p+1/p\sum_{i=2}^{p}\log_{2}i, \end{array}$$
  where the second step follows assuming $\pi^{i}$ is uniform.
3. $C_{\mu }$ = **E**.
4. $T=\sum_{i=1}^{p}\pi^{i}T^{i}=\sum_{i=2}^{p}\pi^{i}\log_{2}i$ bit-symbols.
5. χ=0: This is a bit surprising: No crypticity, no hidden information.

**Figure 14:**
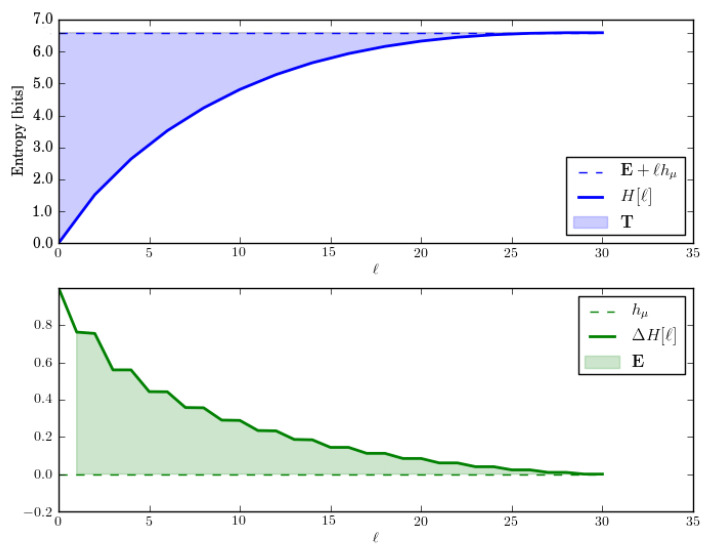
Entropy growth H(ℓ) (**top**) and entropy convergence $h_{\mu }\left(\ell \right)$ (**bottom**) for the Handbag of Necklaces Multistationary Process, as function of word length ℓ=0,…,30 and up to period p=15. (Note that the mixed states were approximated up to Max_Length = 30.).

These are consistent with directly calculating the entropy growth for *M*, as shown in Figure 14.

#### 7.3.2. The Purse Process

The example of two biased coins suggests extending to an infinite number of biased coins in a purse—a bag of coins with different biases. As hinted at in the two-coin case, *all* of the (infinite) complexity is in the mixture and *none* comes from the components.

Moreover, we can choose π to be such that H[π] is finite or infinite. Thus, the Purse Process is an extreme example in which infinite complexity comes from zero-complexity components. There is probably no simpler way to say that a multistationary process is way more than the sum of its (zero complexity) parts.

**Figure 15:**
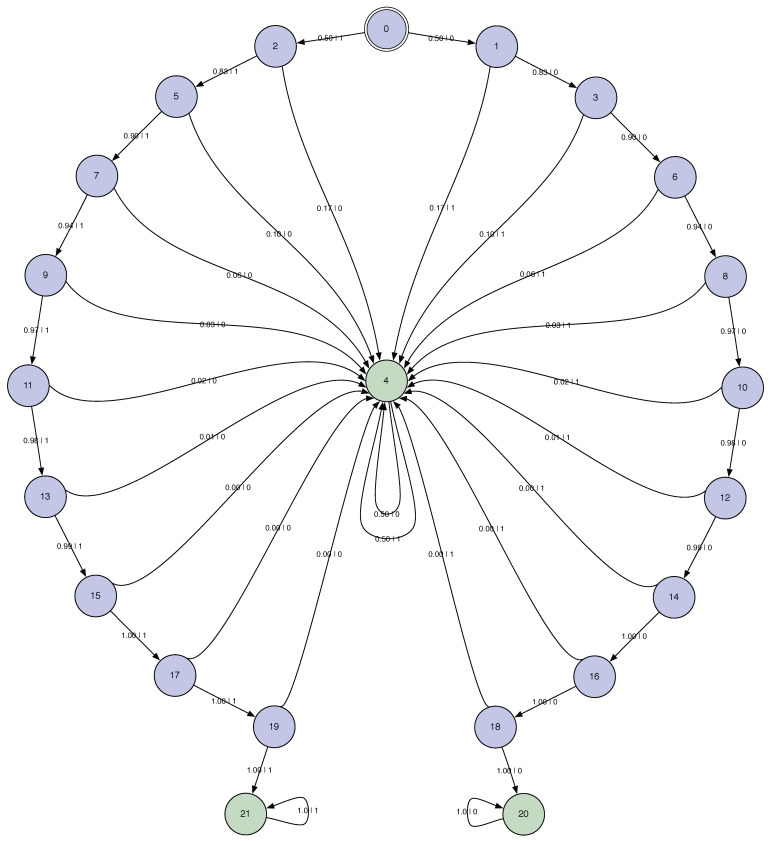
The ϵ-machine HMSP presentation of two completely biased coins and one fair coin—all single-state ϵ-machines: $M=U_{\pi }\left(\mathrm{All}\text{-}1s⨆\mathrm{All}\text{-}0s⨆B\left(\frac{1}{2}\right)\right)$ with $\pi =\left(\frac{1}{3},\frac{1}{3},\frac{1}{3}\right)$.

To obtain a brief sense of the Purse Process, consider an HMSP consisting of three coins of unequal bias and compare this to the case of two coins of Section 7.2.2. Figure 15 shows the HMSP for two completely biased coins and one fair coin. Its basic features were already encountered above. And it suggests a notable generalization to which we now turn.

#### 7.3.3. Mother of All Processes

Finally, consider upping the complexity ante substantially. This generalization is to an HMSP consisting of a mixture of all processes. Let us step through its construction.

First, recall that every stationary process has a unique ϵ-machine presentation. That is, ϵ-machines and stationary processes are in a 1-to-1 correspondence. Second, an efficient algorithm exists to list all ϵ-machines by the number of the recurrent causal states. Reference [34] shows how to systematically enumerate the *ϵ-machine process library* $L_{k}$ for *k*-state ϵ-machines. See Table I there for the list of binary-alphabet topological ϵ-machines. There are 1,117,768,214 such 8-state ϵ-machines.

In the current construction consider only *topological* ϵ-machines for which any branching transitions are taken with fair probability. We refer to each process by its ϵ-machine’s enumeration number—we call this the process’ *Gödel* number.

Second, define the *Process Urn* (PU) as containing the entire library of ϵ-machines. That is, we imagine an HMSP that is the result of reaching into the urn, selecting one ϵ-machine, and having it generate a full realization. The repeatedly sampled PU is a HMSP—*The Mother of All Processes*. Certainly, one of the most nonergodic processes one could work with.

**Definition** **2.** *The* Mother of All Processes *is*

(30) $$M=U_{\pi }\left(\underset{M\in L}{⨆}M\right),$$

*with π being a chosen mixture distribution.*

To simplify, let us examine the HMSP whose components are all one-state and all two-state ϵ-machines. There are now 10 components—three 1-state components and 7 2-state components.

**Figure 16:**
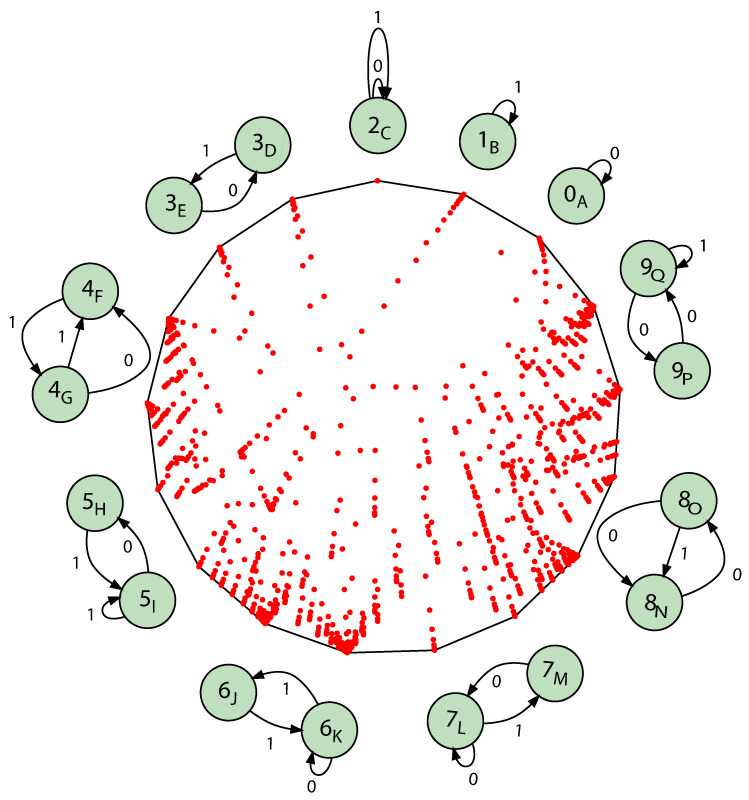
The ϵ-machine presentation of the Mother of All 1-State and 2-State HMSP: $M=U_{\pi }\left({⨆}_{M\in L_{\left\{1,2\right\}}}M\right)$ with $\pi =\left(\frac{1}{10},\ldots ,\frac{1}{10}\right)$. The start state is the 17-vector at $\left(\frac{1}{10},\frac{1}{10},\frac{1}{10},\frac{1}{20},\ldots ,\frac{1}{20}\right)$. The ϵ-machines for each ergodic component are placed around the periphery, dropping the transition probabilities which are either 1 or $\frac{1}{2}$. Their states are aligned to their associated vertex in $\Delta^{17}$. The Fair Coin is the state at the top—the top-most vertex. The mixed states are approximated up to word lengths of ℓ=28.

There are 17 recurrent causal states altogether across the ergodic components. However, the many hundreds of mixed states are no longer usefully presented in a state-transition diagram, as illustrated up to this point. Instead, we plot the mixed states themselves as dots in the simplex $\Delta^{17}$. This is shown in Figure 16. This is a 2D projection in which the recurrent states are the vertices of $\Delta^{17}$ and so appear on its periphery. The start state, with uniform probability across the components and not across the recurrent states, is not in the simplex center.

One notes the concentration of mixed states that move near to $\Delta^{17}$’s vertices, indicating close approaches to synchronization.

There are a number of notable properties, including the following:

1. The simplex vertices correspond to recurrent causal states.
2. There is an uncountably infinite number of transient states. These fill out a complicated fractal measure within the $\Delta^{17}$.
3. All mixed states that are not on vertices are transient states.
4. While it is clear that *M* is not exactly synchronizable [35] as it contains infinite Markov-order components, is it asymptotically synchronizable [36]? What about the synchronizability of approximations to it?

There are a number of open questions, including the following:

1. What is *M*’s statistical complexity dimension $d_{\mu }$ [37]?
2. What is the shortest synchronizing word to go from the Fair Coin to each other ergodic component?
3. How do informational measures grow with word-length approximation?

## 8. Discussion

In addition to their particular application, the ergodic decompositions give important insight into basic questions about what structural complexity is and how to measure it. A number of previous efforts that address these definitional issues consider it a key property that complexity be additive over the components of a system [38]. This is often motivated by a parallel with Boltzmann entropy in thermodynamics. And, for that matter, additivity was also posited as an axiom by Shannon for his measure of surprise [39].

However, the ergodic decompositions here show that the manner in which a system’s components relate to one another—specifically, the mixture distribution—plays a central role in the process’ organization and contributes to quantitative measures of global complexity. The foregoing offered a different, more structural view that goes beyond the ergodic decompositions and statistical mixtures. Constructively, the transient state structure is key to a multistationary process’ global organization and what observations can or cannot reveal.

The lessons here also suggest a skepticism in applying the ergodic decompositions of Section 6. One reason is that underlying them is the assumption of an IID sampling of components, which is not generally valid. Another is that they completely ignore how the internal structures of the components interrelate with each other. And, as shown, this brings out wholly new properties that are not part of any given component nor their sum nor their IID mixture. Indeed, the mixture entropy does not capture this, except in the most limited of cases.

Constructive responses to this will address the new kind of hierarchical structure explicitly represented by the multistationary process’ ϵ-machine transient causal states and their complicated measure in $\Delta^{k}$. Quantitatively, in contrast to the block entropy, entropy rate, and excess entropy, we demonstrated that the transient information is sensitive to this new kind of complexity in structural mixtures. It is this additional structure that makes the organization of multistationary processes way more than the sum of their parts. As a complementary metric, adapting the statistical complexity dimension $d_{\mu }$ suggests itself [37].

## 9. Conclusions

Let us close by exploring several wider implications for thermodynamics, on the one hand, and various attempts to introduce “universal modeling” schemes on the other.

First, we started out highlighting the colloquialism, made familiar by the social movements of the 1960s, that a system is more than the sum of its parts. Presumably, the social reaction then reflected an increasing awareness of the impact of technical systems humans were creating. The preceding development explored in which senses this could be true for truly complex systems—ones consisting of many *structured* components—more akin to the social subsystems than mere atoms. And the various informational ergodic decompositions bolstered the popular understanding.

However, in emphasizing structure and analyzing the concrete process class of hidden multistationary processes, it became abundantly clear—through all of the examples presented—that composite or heterogeneous (to use Gibbs’ word [40]) systems are far more than the sum of their components. Specifically, beyond a mere entropic, missing contribution from increased disorder that arises from the random selection of components, composite systems are markedly more complex. And they are more structured according to the relative interplay of the components’ internal organization. It is that interplay that drives the explosive complexity of multicomponent systems.

On this score, the history of composite systems is perhaps a bit confusing, especially as they arose in the early foundations of thermodynamics. There is, for example, Gibbs’ seemingly contradictory statement, as quoted by Jaynes (p. 13 in [1]), that “The whole is simpler than the sum of its parts”. The ergodic decompositions seemed to say the opposite. However, there is not really a confusion here. First, Gibbs was thinking of the correlations that would emerge between system components when coupled together. Here, we intentionally did not couple the components. Sequels address this. Second, at root, the issue turns on an ambiguous vocabulary for describing randomness and structure. Here, at least, by distinguishing between “randomness” in terms of Shannon’s notion of the flatness of a probability distribution and “structure” in terms of statistical complexity, we shed some light on these important and still evolving issues.

Second, the HMSP construction procedure here gives a rather direct picture of one kind of hierarchical organization in how a stochastic process can be built from other processes. The constructive procedure uses the mixed-state presentation. And this generates a new kind of hierarchy that emerges due to the diverse combinatorial relationships between the components’ internal organizations. Other related hierarchies can be similarly constructed, such as when using generalized hidden Markov models [41] as ergodic components.

Third and finally, modern statistical inference has been treated to a number of formalizations of general learning that make minimal assumptions. Consider, for example, the following:

1. *Universal Priors* [42,43,44]: In the computation-theoretic approach to modeling and statistical inference, there are attempts to define a most-general prior over model space. However, these raise very natural questions: What kind of process would generate such a prior? Moreover, what kinds of difficulties are there in detecting processes drawn according to such a prior?
2. *No Free Lunch Theorem* [45]: This framing makes a number of implicit assumptions about the measure on the Process Urn simplex. Does the theorem hold? Not when you consider structure.
3. *Probably Almost Correct Learning* [46]: This “distribution-free” approach is a bold attempt within machine learning to identify the computational nature of evolution and learning. However, is not this the same thing as assuming any process is possible? If so, then it is analogous to assuming the Mother of All Processes. That is, rather than being “distribution-free” the assumption underlying PAC learning is “distribution-full”.

In light of these, The Mother of All Processes suggests a construction for such assumption-free or minimal-assumption modeling. In this, one is sampling from the space of all processes and exploits the ϵ-machine representation to be specific about probability, on the one hand, and structure, on the other. The realization resulting from this was that the preceding development was able to demonstrate that transient-state structure made explicit the challenges in detecting component processes and that this was captured informationally via the transient information.

## Data Availability

The original contributions presented in this study are included in the article. Further inquiries can be directed to the corresponding author.
